# Dosimetric benefits of half-field arc in prostate cancer treatment

**DOI:** 10.1007/s13246-025-01668-1

**Published:** 2025-11-10

**Authors:** Tamás Ungvári, Döme Szabó, Zsófia Dankovics, Balázs Kiss, Judit Olajos, Károly Tőkési, Georgina Fröhlich

**Affiliations:** 1 Markusovszky University Teaching Hospital, Markusovszky Str. 5, Szombathely, 9700 Hungary; 2 Jósa András University Teaching Hospital, Szent István Str. 68, Nyíregyháza, 4400 Hungary; 3 University of Nyíregyháza, Sóstói Str.31/B, Nyíregyháza, 4400 Hungary; 4 HUN-REN Institute for Nuclear Research, Bem Square 18/C, Debrecen, 4026 Hungary; 5 National Institute of Oncology, Ráth György Str. 7-9, Budapest, 1122 Hungary

**Keywords:** Prostate cancer, Radiotherapy, Treatment planning, Simultaneous integrated boost

## Abstract

The aim of this study is to assess the dosimetric advantages and clinical feasibility of the Half-Field Volumetric Modulated Arc Therapy technique in comparison to conventional Full-Field Arc Therapy and Intensity-Modulated Radiation Therapy for the treatment of prostate cancer. 120 Treatment plans were created for 24 prostate cancer patients using Half-Field, Full-Field, and Intensity Modulated static fields (5-, 7-, and 9-fields). The dosimetric parameters and the homogeneity index were evaluated for the different Planning Target Volumes included pelvic lymph nodes, seminal vesicles, and prostate. Additionally, the dose burden to organs at risk was assessed. The efficiency of the plans was analyzed based on monitor unit usage and the gamma index. Half-Field plans exhibited comparable target coverage to static fields while demonstrating superior homogeneity in comparison to Full-Field plans. This technique resulted in a significant reduction in bladder and rectum doses within the mid- and high-dose ranges, with a V30 for the bladder of 67.8% in Half-Field compared to 75.3% in Full-Field (*p* < 0.001). The Half-Field technique required a significantly fewer monitor units than the Intensitiy-Modulated technique (600.8 vs. 1172.7 for 5-field, *p* < 0.001) resulting in a notable reduction in treatment. Half-Field represents an effective combination of the dosimetric precision of static Intensity Modulated fields with the efficiency of Full-Field arc therapy, offering a promising alternative for prostate cancer treatment. The technique ensures reduced organ at risks doses, enhanced treatment homogeneity and lower complexity, making it a viable option for moderately hypofractionated radiotherapy protocols.

## Highlights

- The Half-Field plan provides better dose homogeneity than the Full-Field plan.
- Half-Field plans reduce bladder and rectum doses in the mid- and high-dose ranges.
- This technique requires fewer monitor units than the Intensity-Modulated technique.
- The half-field technique allows fast and precise treatment.

## Introduction

Prostate cancer is one of the most prevalent malignant neoplasms affecting men and is a significant contributor to the elevated mortality rate observed in this demographic.

Radiotherapy (RT) represents a cornerstone of prostate cancer treatment [1]. RT is performed by a variety of techniques, including three-dimensional conformal RT (3D-CRT), intensity modulated RT (IMRT), volumetric modulated arc therapy (VMAT), stereotactic body radiotherapy (SBRT), stereotactic radiosurgery (SRS), and intensity modulated proton therapy (IMPT), among others [2–5]. The primary objective of all techniques is to deliver the optimal dose to the target volume while minimising the radiation exposure to organs at risk (OAR). The objective of our study is to gain insight into external beam radiation therapy techniques.

The advent of new radiotherapy techniques has enabled the accurate and safe delivery of high doses of radiation, which has been shown to improve tumour control with same level of toxicity compared to lower doses [6].

The available data indicate that the effectiveness of hypofractionated therapy (HFRT) in controlling prostate cancer is comparable to or superior to that of conventional fractionation [7–9]. The reduction in treatment time necessitates fewer visits, which can result in cost savings for the patient and the healthcare system. However, the side effects of HFRT are comparable to those of conventional radiotherapy, although they may be more pronounced in certain instances [10, 11].

Although the VMAT and Simultaneous Integrated Boost (SIB) techniques have been well documented separately, there is a paucity of research on half-field VMAT plans [12], especially in combination with the SIB technique [13]. The aim of this study is to develop a half-field VMAT planning technique that combines the rapidity of VMAT with the improved coverage of IMRT plans, thereby potentially improving the efficacy and safety of radiotherapy treatment of prostate cancer.

This approach is expected to contribute to the advancement of radiotherapy treatment for prostate cancer and to explore new possibilities for the combined use of half-field VMAT and Simultaneous Integrated Boost techniques.

## Methods

A half- and full-field VMAT plan, as well as three IMRT plans (consisting of 5-7-9 fields) have been planned for 24 patients with prostate cancer. The optimisation objectives were identical across all plans. Eclipse v16.1 treatment planning system (Varian Medical Systems, Palo Alto, CA, USA) was used.

The SIB technique was applied: the dose was 22 × 2 Gy for the iliac lymph region, 22 × 2.1 Gy for the seminal vesicles, and 22 × 2.5 Gy for the prostate [14]. A total of 120 plans were created, with half-field plans serving as the reference plans and being compared with the plans generated using alternative methods. The seminal vesicle 6 × 2.1 Gy and prostate 6 × 2.5 Gy boost plans were not included in the study. Instead, the large field plans were examined from a dosimetric perspective.

The contours of the rectum and bladder were generated using the Siemens SOMATOM Go.Sim CT simulator software (Syngo.via VA40, Siemens Erlangen, Germany). A CT series was acquired with a slice thickness of 3 mm. The contours were approved by radiation therapy technicians (RTTs), medical physicists and radiation oncologists. The VMAT plans and the 5i IMRT plan were performed with a collimator angle of 0 degrees in the 5i plan, with the gantry at 260, 340, 20, 100, and 180 degrees. In the 7i and 9i plans, the collimator varied between 86 and 94 degrees.

The following dose-volume parameters were calculated and compared in HF- and FFVMAT, 5i-, 7i- and 9i IMRT plans:

D2—the minimal dose received by the 2% volume of the PTV (Gy),

D98—the minimal dose received by 98% volume of PTV (Gy),

D100—the minimal dose received by the 100% volume of the PTV.

HI—homogeneity index:

1 $$ HI = \frac{{\left( {D2\% - D98\% } \right)}}{D100}*100 $$

The homogeneity index (HI) values indicate that HFVMAT can provide a more uniform dose distribution within the Planning Target Volume (PTV), potentially enhancing tumor control and reducing the risk of hot spots that could lead to toxicity [3, 12].

V38Gy—The area covered by the 38 Gy curve was selected to carry out a comparative analysis of the plans. It was assumed that negligible differences were observed for amounts below 30 Gy. Conversely, for volumes above 40 Gy, the dose is closer to the lymphatic region, which may result in smaller differences between the plans. In terms of irradiated volume of 38 Gy, this could result in a notable elevation of the risk of adverse side effects.

Gamma index values were used to detect the differences between the planned and delivered doses [15–22]. All techniques were investigated for each plan, and the gamma index was analyzed across techniques.

The Arccheck phantom and software version 6.2.6 (Sun Nuclear Corporation, Melbourne, FL, US) were used with settings of 2 mm and 2% and a 10% threshold to measure the gamma index. A higher gamma index indicates a greater degree of agreement between the planned and delivered doses.

Shapiro–wilk test was used to detect the distribution of the variables. To ascertain the discrepancies between the techniques, Friedman analysis of variance and Fisher LSD test was employed in Statistica v12.5 (StatSoft, Tulsa, OK, USA). The *p* = 0.05 was the significance level.

## Results

### Dose-volume parameters of the pelvic lymph node (PLN) region)

The D2 value for the VMAT plans was 45.6 Gy, representing a reduction of approximately 2.2% in comparison to the IMRT plans (46.2 Gy). With regard to D98, the HFVMAT exhibited a 2.6% reduction in coverage relative to the IMRT plans (*p* < 0.001) while demonstrating a 7.5% improvement in comparison to the FFVMAT (D98 *p* < 0.001).

In the pelvic lymph node (PLN), the results of HFVMAT (45.6 Gy) and FFVMAT (45.6 Gy) doses were similar in terms of observed dosimetric results.

Nevertheless, the 5-, 7-, and 9-field IMRT techniques exhibited slightly higher values (46.1 Gy, 46.2 Gy, and 46.2 Gy, respectively).

The minimum doses of PLN region exhibited by the HFVMAT (41.9 Gy) and FFVMAT (39.2 Gy) were found to be lower than those observed in the IMRT plans, with values ranging from 42.8 to 43.0 Gy. In terms of the D100, HFVMAT (37.6 Gy) and FFVMAT (34.7 Gy) also yielded lower values than all of the IMRT plans (37.2–37.7 Gy).

The HFVMAT demonstrated superior HI compared to the FFVMAT, although the IMRT plans exhibited even more homogeneous distributions. See Table 1.

**Table 1:** Beam configurations for each planning technique, including gantry angles and collimator settings, are as follows: HFVMAT plans utilized dual half-fields, whereas FFVMAT plans employed a full-field approach. IMRT plans were created using 5, 7, or 9 static fields

Technique	Gantry angles	Collimator angle(s)
HFVMAT	field1 0.0 -179 ClockWise; field2 179–181 CounterClockWise; field3 181–0 ClockWise	all field 0°
FFVMAT	field1 0.0 -179 Clock Wise; field2 179–181 CounterClockWise; field3 181–0 ClockWise	all field 0°
5i IMRT	field1 260°, field2 340°, field3 40°, field4 100°, field1170°	all field 0°
7i IMRT	field1 180°, field2 125°, field3 70°, field4 20°, field5 340°, field6 290°, field7 235°	field6 94° all others 90
9i IMRT	field1 160°, field2 120°, field3 70°, field4 40°, field5 0°, field6 320°, field7 290°, field8 200°, field9 240°	field1 86°, field2-field7 90°, field8 94°, field9 90°

### Dose-volume parameters of the prostate-seminal vesicle (PSV)

With regard to the D98 parameter, all plans, with the exception of the FFVMAT plan, achieved the prescribed dose. The D100 exhibited a minimal difference (1.3%) between the HF and IMRT plans (*p* > 0.5). The D100 value for FFVMAT is already 2.3%. With the exception of the FFVMAT plan, which exhibited an HI value of 25.7 on average, the HI values are comparable. No significant discrepancy was observed between the D2 values.

It was observed that the HFVMAT (56.7 Gy) and FFVMAT (56.86 Gy) exhibited comparable outcomes, whereas the IMRT plans (57.3–57.6 Gy) demonstrated slightly elevated values.

The HFVMAT (46.2 Gy) and FFVMAT (44.9 Gy) exhibited lower values than the IMRT plans (46.8–47.4 Gy) with regard to D98.

With respect to D100, the HFVMAT (40.9 Gy) and FFVMAT (39.59 Gy) exhibited comparable values, whereas the IMRT plans (40.15–41.0 Gy) demonstrated slightly higher values.

### Dose-volume parameters of the PTV of Prostate (PRS)

The prescribed dose (55 Gy) was most closely approximated by D98 of IMRT plans (53.46 Gy) 2.8%, with a deviation of 4.2% for D98 of HFVMAT and 7% for FFVMAT. These values were found to be below the IMRT value of 53.5 for the D98 parameter. The D2 value, which represents an overdose for VMAT plans, is comparable to that of IMRT, with an average of 57.5. The discrepancy is 1.2%.

D2: The maximum doses delivered by the HFVMAT (56.7 Gy) and FFVMAT (56.8 Gy) were found to be comparable. The IMRT techniques had maximum doses of 57.6 Gy, 57.49 Gy and 57.3 Gy, respectively, for the prostate target volume at the prescribed 55 Gy.

D98: The minimum dose observed for the HFVMAT was 52.4 Gy, which was higher than that observed for the FFVMAT, which was 51.26 Gy. Nevertheless, even higher doses were observed for IMRT techniques, with values of 53.6 Gy, 53.4 Gy, and 53.4 Gy, respectively. The lowest dose observed was D100. The minimum doses observed for HFVMAT (47.9 Gy) and FFVMAT (46.4 Gy) were found to be lower than those achieved by IMRT techniques (49.6 Gy, 49.1 Gy, 49.3 Gy), which resulted in higher doses.

The HI were found to be similar, with IMRTs ranging from 22.0 to 22.7. There is a significant difference between the HI values of HFVMAT and FFVMAT 22.6 vs 25.7 (*p* < 0.001).

Our results are summarised in Table 1.

### Quality assurance

For quality assurance, the planned and delivered doses were evaluated using a gamma index. The analysis revealed that the HFVMAT technique achieved the highest average gamma index value (98.2 ± 1.31), followed by FFVMAT (97.45 ± 1.29), and the IMRT techniques: 5-field (97 ± 1.38), 7-field (96.7 ± 1.55), and 9-field (96.88 ± 1.25).

Higher gamma index values indicate a better match between the planned and delivered doses. The results demonstrate that HFVMAT provides superior dose agreement. While no statistically significant difference was observed between HFVMAT and FFVMAT (*p* = 0.1610), significant differences were found when comparing HFVMAT with IMRT plans: HFVMAT vs. 5i (*p* = 0.011), HFVMAT vs. 7i (*p* = 0.002), and HFVMAT vs. 9i (*p* = 0.003).

#### Dose-volume parameters of the bladder

V30: The HFVMAT (67.8%) demonstrated a lower dose compared to the FFVMAT (75.49%), while the IMRT techniques (60.15–62.9%) exhibited superior performance.

V40: The HFVMAT (26.16%) also demonstrated a lower dose compared to the FFVMAT (31.1%), while the IMRT plans (28.2–29.15%) exhibited results that were nearly identical.

**Fig. 1:**
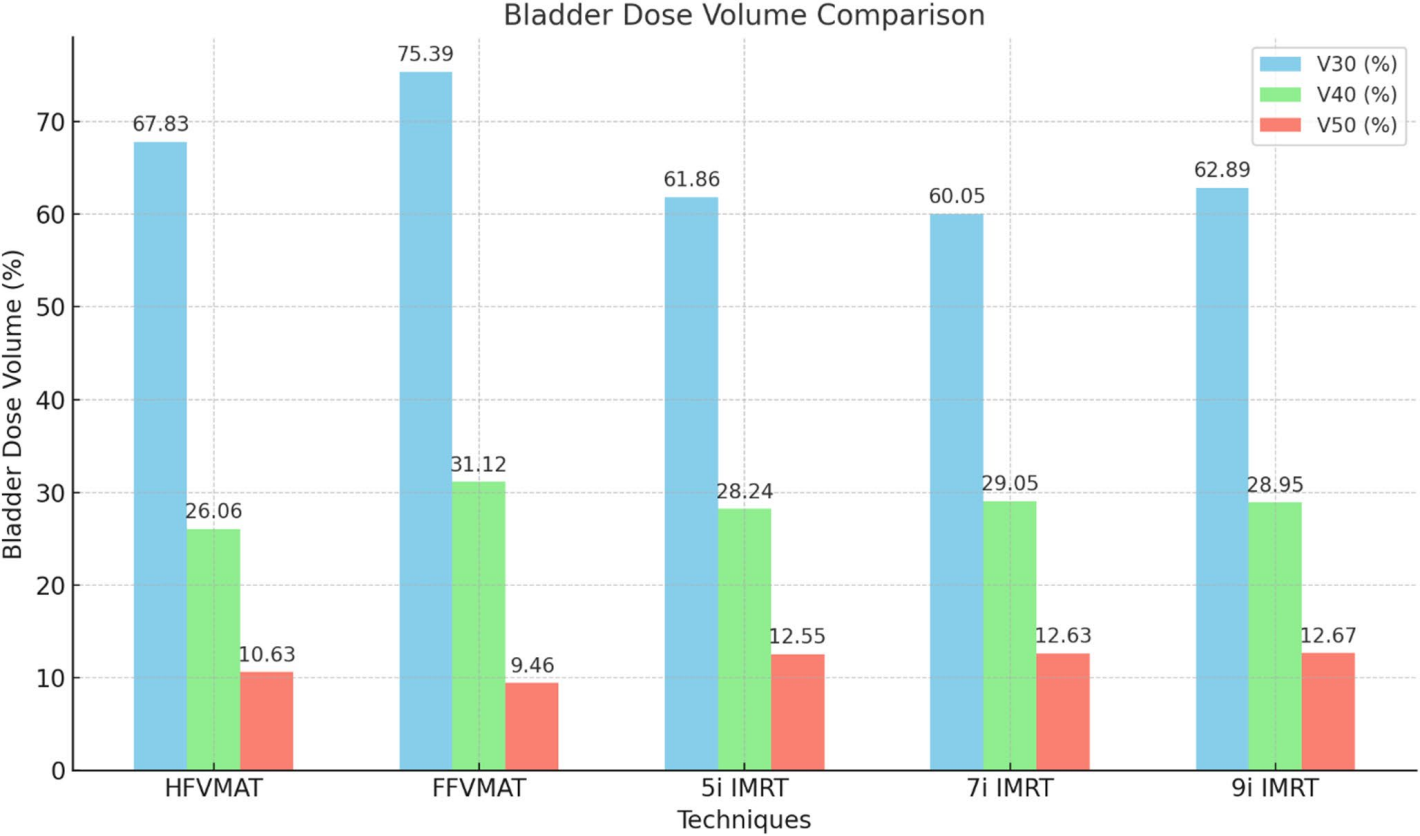
The V30, V40 and V50 dose-volume parameters of the bladder in the HFVMAT, FFVMAT 5i-, 7i- and 9i IMRT treatment plans of the prostate

V50: The HFVMAT exhibited higher values than the FFVMAT (10.6% vs. 9.5%), while the IMRT plans demonstrated the highest values (12.65–12.7%). Figure 1 shows the bladder doses.

#### Dose-volume parameters of the rectum

With regard to the V30 parameter, the values obtained for the HFVMAT and FFVMAT were found to be similar. In contrast, the IMRT plans yielded higher values, with a range of 63.4% to 67.25%.

V40: The results for the HFVMAT (24.8%) and FFVMAT (29.0%) were also found to be nearly similar, while the IMRT plans (25.3–26.5%) exhibited slightly higher values.

**Fig. 2:**
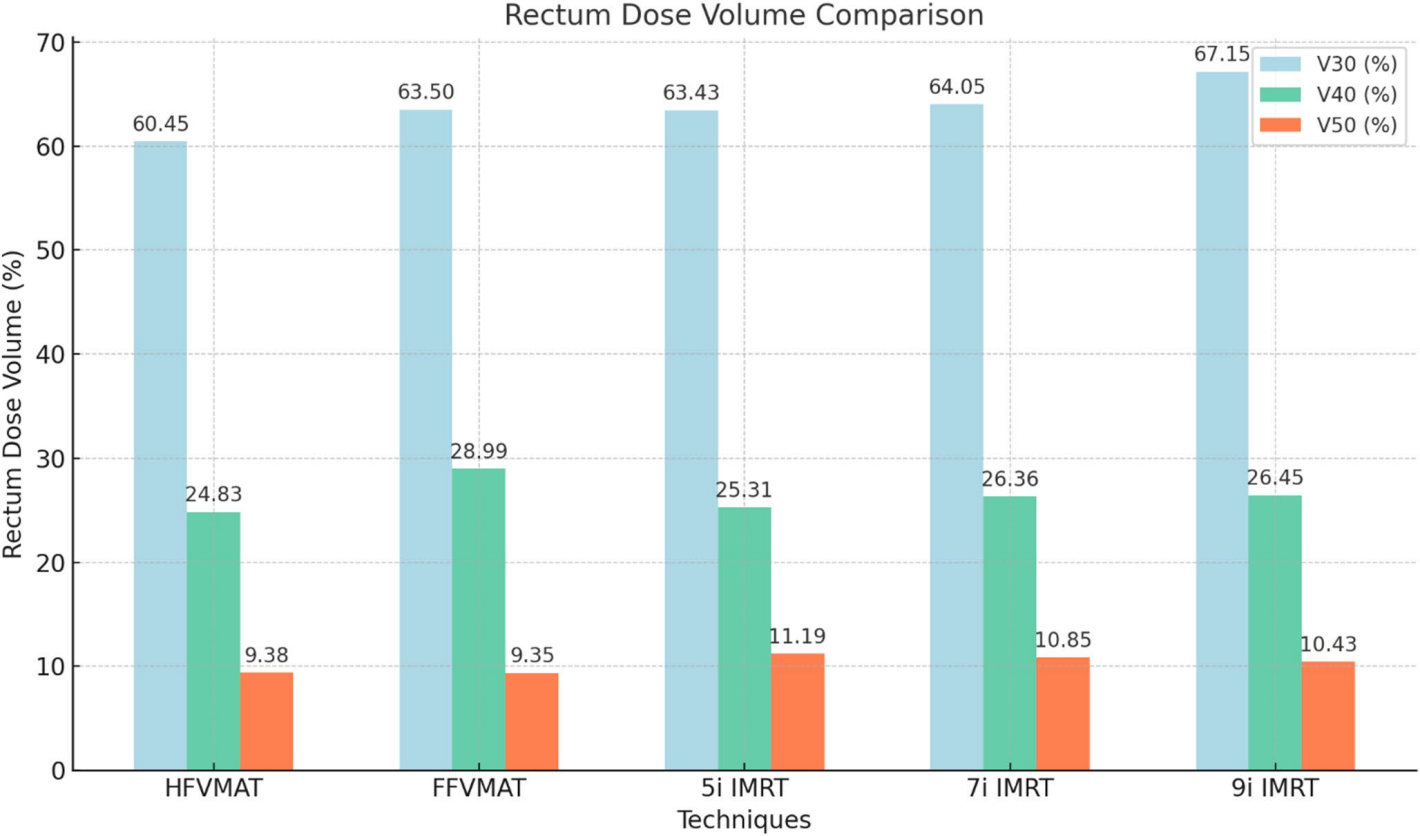
The V30, V40 and V50 dose-volume parameters of the rectum in the HFVMAT, FFVMAT 5i-, 7i- and 9i IMRT treatment plans

V50: The HFVMAT (9.4%) and FFVMAT (9.45%) exhibited comparable values, whereas the IMRT plans (10.4–11.2%) demonstrated higher values (Fig. 2).

The volume of the 38 Gy is as follows: For both the HFVMAT and FFVMAT, the 38 Gy covered volumes were lower compared to the IMRT plans. Specifically, the HFVMAT emitted 1282.1 cm3, while the FFVMAT produced 1364.9 cm3. The volumes covered by the IMRT plans ranged from 1542.1 to 1664.2 cm^3^. A comparison of the HF- and FFVMAT plans with the IMRT plans reveals a significant discrepancy in the protection of normal tissue (V38Gy). (*p* < 0.001).

The cumulative monitor units (MU) required for the HFVMAT and FFVMAT were significantly lower than those required for the IMRT plans, with values of 600.8 MU and 390.3 MU, respectively. The results of the gamma analysis demonstrated that the HFVMAT exhibited a higher degree of compliance than the FFVMAT and IMRT plans, with a compliance rate of 98.1% compared to 97.3% and 96.8% to 97.0%, respectively.

### Summary of statistics

A significant difference was observed between the HFVMAT group and the others group in PTVs, Rectum, Bladder, Total MU and Gamma index parameters (See Table 2). The differences are highlighted.

**Table 2:** The dose-volume parameters (mean value and ± standard deviation) of the HFVMAT

PTV/Organ	HFVMAT	FFVMAT	5i	7i	9i	*p* value
PLN D2	45.60 ± 0.65	45.59 ± 0.59	46.13 ± 0.78	46.18 ± 0.75	46.19 ± 0.84	0.017
PLN D98	41.91 ± 0.74	39.16 ± 1.20	42.77 ± 0.75	43.12 ± 0.72	43.10 ± 0.80	< 0.001
PLN D100	37.61 ± 1.59	34.66 ± 1.76	37.18 ± 2.47	37.93 ± 2.88	37.97 ± 3.09	< 0.001
PLN HI	8.37 ± 2.03	14.62 ± 3.43	7.64 ± 1.41	6.96 ± 1.24	7.018 ± 1.064	< 0.001
PSV D2	56.65 ± 0.54	56.75 ± 0.34	57.60 ± 1.12	57.35 ± 0.91	57.34 ± 1.00	0.013
PSV D98	46.20 ± 1.99	44.87 ± 2.48	47.42 ± 2.70	46.96 ± 2.57	46.84 ± 2.54	< 0.001
PSV D100	40.94 ± 1.82	39.48 ± 2.30	40.94 ± 2.97	40.28 ± 3.25	40.04 ± 3.28	< 0.001
PSV HI	22.61 ± 4.19	25.73 ± 5.26	22.04 ± 5.48	22.50 ± 5.19	22.72 ± 5.091	< 0.001
PRS D2	56.73 ± 0.53	56.79 ± 0.33	57.64 ± 1.11	57.39 ± 0.90	57.31 ± 1.00	0.033
PRS D98	52.35 ± 1.16	51.15 ± 1.09	53.56 ± 1.08	53.41 ± 0.83	53.42 ± 0.86	< 0.001
PRS D100	47.91 ± 2.94	46.35 ± 2.75	49.56 ± 2.93	49.08 ± 2.92	49.33 ± 2.66	< 0.001
PRS HI	7.95 ± 1.81	10.24 ± 2.19	7.41 ± 1.35	7.23 ± 1.43	7.08 ± 1.51	< 0.001
Bladder V30	67.82 ± 8.59	75.38 ± 9.93	61.85 ± 5.57	60.05 ± 9.67	62.88 ± 8.23	< 0.001
Bladder V40	26.06 ± 9.317	31.11 ± 5.23	28.24 ± 8.77	29.05 ± 9.20	28.95 ± 9.20	< 0.001
Bladder V50	10.62 ± 7.63	9.46 ± 6.83	12.55 ± 8.09	12.63 ± 8.11	12.67 ± 8.09	< 0.001
Rectum V30	60.45 ± 5.20	63.50 ± 5.94	63.43 ± 6.26	64.05 ± 7.33	67.14 ± 9.41	< 0.001
Rectum V40	24.82 ± 7.18	28.98 ± 6.70	25.30 ± 6.59	26.35 ± 5.88	26.45 ± 5.97	< 0.001
Rectum V50	9.37 ± 4.21	9.34 ± 4.43	11.19 ± 5.19	10.85 ± 5.05	10.42 ± 5.11	< 0.001
V38 Gy	1282.04 ± 265.98	1364.66 ± 241.950	1542.12 ± 366.92	1612.27 ± 407.99	1664.18 ± 444.59	< 0.001
total MU	600.75 ± 58.41	390.33 ± 31.76	1172.70 ± 100.52	1480.79 ± 130.44	1664.25 ± 151.47	< 0.001
Gamma index	98.12 ± 1.31	97.44 ± 1.28	97.00 ± 1.38	96.70 ± 1.54	96.87 ± 1.25	< 0.001

FFVMAT. 5i, 7i, and 9i plans

PLN: Pelvic lymph node region PTV

PSV: Seminal vesicles PTV

PRS: Prostate PTV

V30: the volume of the risk organ that receives at least 30 Gy of doses

V40: the volume of the risk organ that receives at least 40 Gy of doses

V50: the volume of the risk organ that receives at least 50 Gy of doses

## Discussion

HFVMAT represents a novel approach in prostate cancer radiotherapy, integrating the speed of VMAT with the dosimetric advantages of IMRT. This study demonstrated that HFVMAT achieves comparable target volume coverage to IMRT while significantly improving dose homogeneity over FFVMAT. Additionally, HFVMAT effectively reduces radiation exposure to the bladder and rectum and requires fewer monitor units, potentially optimizing treatment efficiency and minimizing intrafraction motion.

HFVMAT exhibited a comparable target dose distribution to IMRT while achieving significantly better dose homogeneity than FFVMAT.

Remarkable differences were observed in PLN coverage, PSV, PRS coverage and rectal bladder burden. In terms of planning target volume coverage, HFVMAT is little or not lagging behind IMRT plans (Table 3).

 In Table 3, bold italics indicate parameters with a statistically significant difference (p < 0.05).

**Table 3:** The results of the post-hoc tests in the comparison of the dose-volume parameters of HFVMAT vs FFVMAT 5i-, 7i- and 9i IMRT treatment planning techniques

	FFVMAT	5i	7i	9i
D2 PLN	0.0978	***0.0135***	***0.0099***	***0.0061***
D98 PLN	** < *0.001***	** < *0.001***	** < *0.001***	** < *0.001***
D100 PLN	** < *0.001***	0.5493	0.5832	0.6094
HI PLN	** < *0.001***	*0.2189*	***0.0210***	***0.0232***
D2 PSV	0.6680	** < *0.001***	***0.0068***	***0.0054***
D98 PSV	0.0646	0.0920	0.3390	0.3743
D100 PSV	0.0725	0.9954	0.4195	0.2684
HI PSV	***0.0358***	0.6978	0.9784	0.9426
D2 PRS	0.8039	** < *0.001***	***0.0099***	***0.016***
D98 PRS	** < *0.001***	** < *0.001***	** < *0.001***	** < *0.001***
D100 PRS	0.0601	***0.0456***	0.1084	0.0843
HI PRS	** < *0.001***	***0.0175***	***0.0029***	***0.0484***
Rectum V30	0.1344	0.1430	0.1023	***0.0012***
RectumV40	***0.0285***	0.0797	0.4826	0.3870
RectumV50	0.9833	0.1948	0.3566	0.4508
Bladder V30	***0.0028***	***0.0175***	***0.0029***	***0.0484***
Bladder V40	***0.0411***	0.3751	0.3031	0.2398
Bladder V50	0.0603	0.3931	0.4739	0.3642
V38 Gy	0.4182	***0.0118***	***0.0034***	** < *0.001***
Total MU	** < *0.001***	** < *0.001***	** < *0.001***	** < *0.001***
Gamma index	0.161	***0.011***	***0.002***	***0.003***

In Table 3, bold italics indicate parameters with a statistically significant difference (p < 0.05)

One of the most notable advantages of HFVMAT is its ability to reduce bladder and rectum doses. Our results show that HFVMAT achieves lower mid- and high-dose volumes in these OARs compared to FFVMAT. The bladder V30 was significantly reduced (67.8% in HFVMAT vs. 75.3% in FFVMAT, *p* < 0.001), and similar improvements were observed in the rectum V30 values. Compared to IMRT, HFVMAT demonstrated comparable or slightly improved OAR sparing, suggesting that it provides a clinically meaningful reduction in toxicity risks [5, 7, 14].

The HFVMAT technique resulted in the smallest volume of tissue exposed to 38 Gy (1282.05 cm^3^), followed by FFVMAT (1364.90 cm^3^). In contrast, the IMRT techniques exposed progressively larger volumes: 5-field IMRT (1542.12 cm^3^), 7-field IMRT (1612.28 cm^3^), and 9-field IMRT (1664.18 cm^3^). This indicates that HFVMAT provides the most effective sparing of surrounding tissues at this dose level compared to the other techniques.

**Fig. 3:**
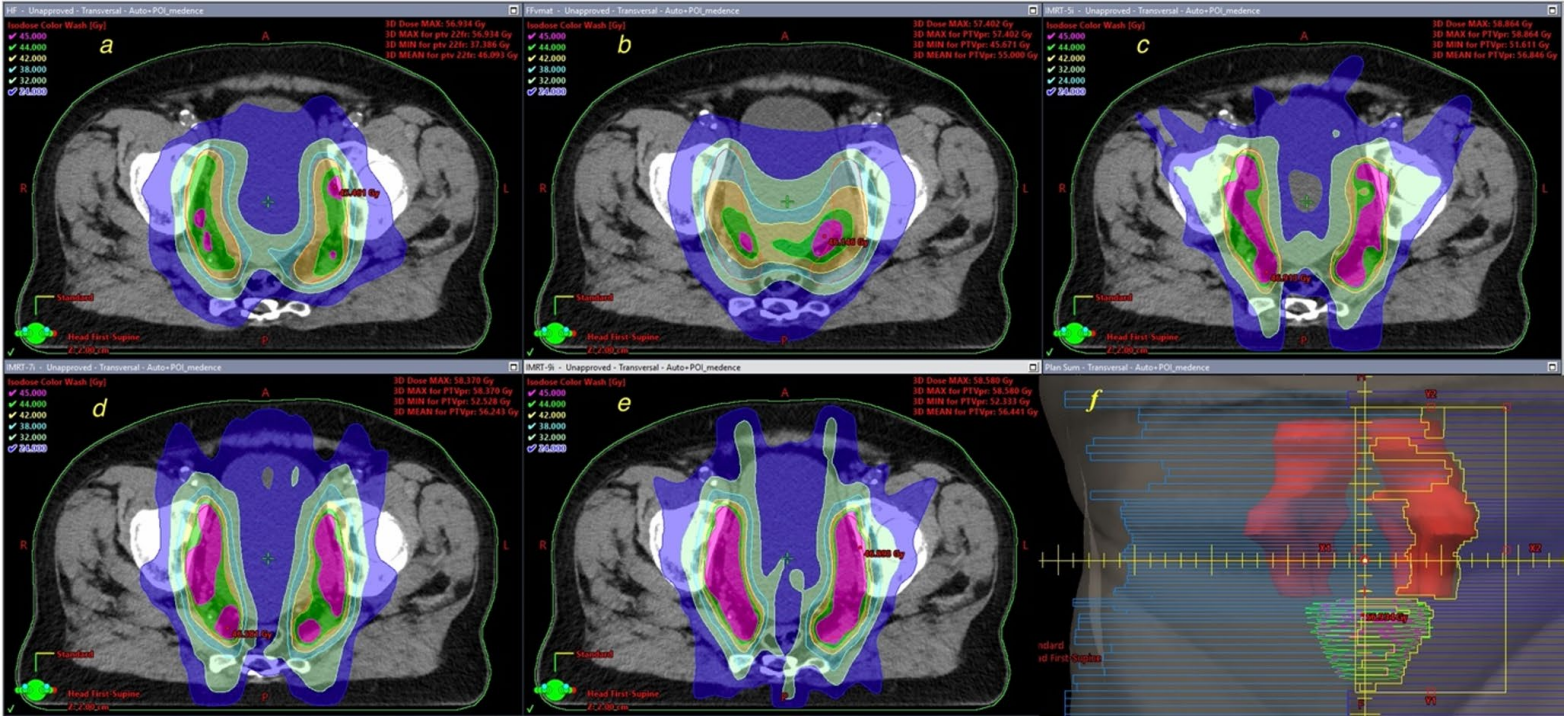
Dose distribution in an axialCT slice. **a** HFVMAT, **b** FFVMAT, **c** 5i IMRT, **d** 7i IMRT, **e** 9i IMRT, **f** Beams Eye View of one field of HFVMAT

HFVMAT required significantly fewer monitor units (MU) compared to IMRT (600.8 vs. 1172.7 for 5-field IMRT, *p* < 0.001), indicating a reduction in radiation treatment time. This reduction in MU not only shortens treatment duration, but may also improve patient comfort and reduce intrafractional motion, which may result in more accurate dose delivery and improve feasibility of plans [12, 16, 19, 23]. Dose distribution of the different planning techniques can be seen on Fig. 3.

The results of gamma index demonstrate that HFVMAT exhibits greater robustness and reduced susceptibility to leaf movement in comparison to FFVMAT and IMRT techniques. The degree of concordance between the plan and the measurement was 98.1%, with the lowest value being 96.7% for the 7i.

Our findings align with prior research on VMAT and IMRT techniques, but few studies have specifically evaluated half-field VMAT in the context of prostate cancer. Jang et al. reported similar benefits in dose homogeneity and OAR sparing with a half-beam approach in pelvic irradiation, but their study did not explore the application of the SIB technique [24]. Similarly, Yu et al. highlighted the advantages of half-beam VMAT in gynecological cancers, supporting the potential of this technique in complex treatment scenarios [12].

The HFVMAT plan can be integrated into automated workflows [25, 26].

Our results are consistent with the research of Pei Chieh Yu et al. HFVMAT is not only a conventional dosage and total female pelvic irradiation but also for SIB dosing of prostate radiotherapy [12].

## Conclusion

HFVMAT presents a viable alternative to IMRT and FFVMAT for prostate cancer treatment, combining the speed of VMAT with the dosimetric precision of IMRT. The technique demonstrates superior dose homogeneity, reduced OAR exposure, and enhanced treatment efficiency. Given these advantages, HFVMAT could be a valuable addition to modern radiotherapy protocols, particularly for hypofractionated treatment regimens. Further clinical validation is warranted to confirm its long-term benefits.

## Declarations

### Data availability

The data that support the findings of this study are available from the corresponding author upon reasonable request.
